# Mind the Mother When Considering Breastfeeding

**DOI:** 10.3389/fgwh.2020.00003

**Published:** 2020-09-15

**Authors:** Veronica Rivi, Greta Petrilli, Johanna M. C. Blom

**Affiliations:** ^1^Department of Biomedical, Metabolic and Neural Sciences, University of Modena and Reggio Emilia, Modena, Italy; ^2^Unità di Psichiatria-Ambulatorio Varenna per la Salute Mentale Perinatale, ASST Papa Giovanni XXIII, Bergamo, Italy; ^3^Area Perinatale SIPRe-Milano, Società Italiana di Psicoanalisi della Relazione, Milan, Italy; ^4^Centre of Neuroscience and Neurotechnology, University of Modena and Reggio Emilia, Modena, Italy

**Keywords:** maternal mental health, breastfeeding, perinatal period, screening, risk factors

## Introduction

Depression and anxiety disorders represent the most common obstetric complications during pregnancy and the first-year post-partum, reducing the mother's ability to effectively perceive, decipher, and respond to their infant needs (1). An important question faced by mothers is whether to breastfeed their newborn child. Maternal mental health and breastfeeding have a tight but mixed relationship. Contrasting results have been reported in the literature (2): for each study demonstrating that breastfeeding is protective for the onset of anxiety and/or depression (3), there is a study showing that the difficulties related to breastfeeding can exacerbate the incidence and/or severity of maternal psychiatric illness (4, 5). Similarly, while several studies demonstrate a negative impact of depression on the duration of breastfeeding (6–8), others show that postpartum mental illness could emerge as consequence of the early interruption of breastfeeding (9). Moreover, the all or none view that “breast is best” established by our society, makes some women feel inadequate if they choose not to breastfeed (10). Nevertheless, feeling inadequate or ashamed is not equivalent with suffering from anxiety or postpartum depression (PPD), and some women do not feel any negative pressure if they decline to breastfeed. There is no explicit reason, if a woman does not want to breastfeed, that her mental health and well-being depends on this decision or that she will experience any negative outcome as a result of her decision (9, 11). Given this and considering that breastfeeding recommendations are a critical and complex topic in public health discussions, our aim is to highlight various aspects surrounding breastfeeding with the purpose to propose to stably include screening for mental health in programs tailored to the mother.

## Breastfeeding as Moderator

Considering the numerous and complex biological, physiological, psychological, environmental, and sociocultural factors involved, breastfeeding can be defined at best as a moderator, decreasing or increasing anxiety, and depressive symptoms depending on the breastfeeding intentions of the mother (9, 11, 12), her personal history, and any pre-existing or current maternal mental health and treatment related issues (2, 13). Each (breast)feeding experience, in fact, should be considered “with the mother in the mind,” assuming that there is not a one-size-fits-all relationship between breastfeeding and maternal mental health. Successful breastfeeding starting immediately after birth and its continuation for the recommended or desired period is not the exclusive responsibility of the mother. It is a shared collective responsibility, with many stakeholders and moderating factors, among which the sociocultural environment, the context (family, workplace, healthcare system), and maternal physical and mental health (13–15).

Research routinely suggests that antenatal depression, considered in isolation, negatively influences breastfeeding. However, the effect of antenatal depression on breastfeeding should not be considered as a separate entity but is the result of many factors, such as the psychosocial and socio-economic context of the pregnant woman (11). Therefore, if we want to truly understand the relationship between antenatal anxiety and depression and breastfeeding, we should embrace a multidimensional approach to better understand the effects of the factors driving the intention to breastfeed which is set off by the mothers' state of mind. The main challenge is not to understand the role of each factor and context but the relationships and connectivity among them. New conceptual thinking will result in increasingly explanatory and predictive models which may offer a more realistic image of the strengths and vulnerabilities of pregnant women who intend to breastfeed by stressing the dynamic nature of these relationships. This type of approach may have important implications for clinical practice as it not only indicates which domains mostly influence antenatal depression, but also which factors are most central to the development of antenatal depression, and, therefore, may direct its overall effect on breastfeeding. As a consequence, we will be able to better recognize interrelated behaviors allowing to isolate the domain(s) most central to overall psychosocial risk, thus, improving successful breastfeeding while developing programs fundamental for the screening, surveillance and monitoring of antenatal depression and mental health more generally.

## Impact of General Policy

Furthermore, it is important to be mindful that the policy of public health organizations, like the WHO, issued important recommendations promoting the concept that “breast is best” is based mainly on the need of third world countries for nutrition, and not on emotional well-being (16). Consequently, this policy is currently questioned extensively and a more inclusive belief of “fed is best” is put forward, involving women who breast-feed, bottle-feed or both and which stresses the first and foremost importance of providing nutrition avoiding at all cost, infant undernutrition. While more inclusive this approach still lacks a focus on the emotional well-being of the mother.

## The Benefits and Potential Harms of Breastfeeding

Breastfeeding is more than just the delivery of the optimal infant nutrition, and offers a wide range of psychological benefits for both the infant and the mother, providing one of the earliest opportunities to facilitate maternal awareness of her infant needs and vice versa (17). The act of breastfeeding, in fact, promotes hormonal processes that induce the release of oxytocin, an important hormone related to maternal bonding, and attenuates the cortisol response to stress which when consistently high, is one of the strongest risk factors for the development of psychiatric disorders (17). Moreover, breastfeeding supports the regulation of sleep and wake patterns for both mother and infant and sustains and improves maternal self-efficacy (1). Consequently, breastfeeding mothers are more likely to report positive mood, less anxiety, and increased calm compared to formula feeding mothers (1, 8). Beyond the psychological benefits, breastfeeding provides substantial nutritional, cognitive, emotional, and immunologic benefits for the infants and their mothers. Breast milk, in fact, not only gives infants all the nutrients required for healthy development, but it also contains antibodies that protect infants from common childhood pathologies. Moreover, breastmilk affects the buildup of viral populations in the gut of the neonate which confers protection against harmful viruses and is thus fundamental in the interaction between the neonate and the microbial environment (18). For mothers, breastfeeding has proven to confer lower risk of breast and ovarian cancer, greater postpartum weight loss and decreased blood pressure compared with no breastfeeding (19).

In contrast, breastfeeding, which requires sustained mother–infant contact could represent a serious challenge for those mothers who suffer from perinatal mental diseases. Consequently, depressed mothers often report lower breastfeeding self-efficacy and tend to breastfeed less or for a shorter period of time (2, 6, 9).

Even though women suffering from depression and anxiety during the perinatal period tend to be less likely to initiate or to maintain breastfeeding (3, 7, 9), this does not preclude the possibility that breastfeeding has a positive effect on depressive and/or anxiety related symptoms during this period. In fact, some women who had no intention to breastfeed during gestation but changed their minds and attempted to breastfeed after all, showed a significant decrease in depressive symptoms from childbirth to 3 months postpartum (2, 9). Interestingly, other studies reported that mothers who do not suffer from anxiety and/or depression during pregnancy and do not initiate or maintain breastfeeding, are more at-risk for depression and/or anxiety in the postpartum period (9).

Also, breastfeeding has severe psychological consequences in women who were not depressed before delivery and intended to breastfeed but were not able to do so (10). Together these results are an ulterior demonstration of the complex and heterogeneous effects of breastfeeding on maternal mental health and suggest important implications for the way in which these mothers should be supported.

Although maternal depression and anxiety have been identified as risk factors for early breastfeeding cessation (3, 7), some studies showed that instead depression and anxiety could result as consequence of breastfeeding interruption (1, 2). Women who experience depression/anxiety and then proceed to breastfeed, may or may not experience further anxiety or depression even if they had early negative breastfeeding experiences. Thus, it is possible that mothers may have had an early negative breastfeeding experience (e.g., difficulty with latch-on, perceived insufficient milk, etc.) (4, 10) but have overcome these difficulties, with adequate support, and have moved forward to continue to breastfeed and in the end had a positive breastfeeding experience (successful latch-on, adequate or increased milk supply, etc.) accompanied by positive mental health outcomes (pride, increased breastfeeding self-efficacy, improved mood) as a result (4, 9).

## Operating “With the Mother in the Mind”

Working “with the mother in the mind” also means to consider previous and current maternal life experiences (i.e., traumatic episodes, antidepressant treatments, age, parity, marital status, family income, social support, or maternal employment…) as significative factors influencing the perception of motherhood. In fact, when deciding whether to breastfeed, each new mother is influenced not only by her physical and psychological ability and personal beliefs, but also by social and cultural customs and norms. In those instances, in which breastfeeding improves maternal mental health this practice should be encouraged but when breastfeeding negatively affects maternal psychiatric symptoms, mothers should not be left to feel guilty if they choose to seek alternative forms of feeding. Given this, it is important that their partner and other family members participate in the decision related to the risks and benefits of breastfeeding and share the necessity to consider alternative options. Such considerations and inclusiveness could have a substantial positive impact on maternal mental health and may prevent further complications and negative outcomes for both the mothers and their children.

Maybe now is the moment we should look for a better more inclusive and multidisciplinary approach to the expectations, preferences, and decision-making of women regarding breastfeeding and revisit norms and recommendations that dominate conversations between the care team and women who are trying to decide how to feed their baby. First, and most importantly, the “breast is best” approach does not reflect the preference of all women and does not uniquely confer mental well-being. Also, many women resent that alternatives to breastfeeding are negatively framed which undermines confidence in them, to make the best choice for herself as well as for her baby. Instead, mothers prefer possibilistic, optimistic, or encouraging messages, ones that acknowledge and respect their choice and even anticipate the possibility of good outcomes.

**Figure 1:**
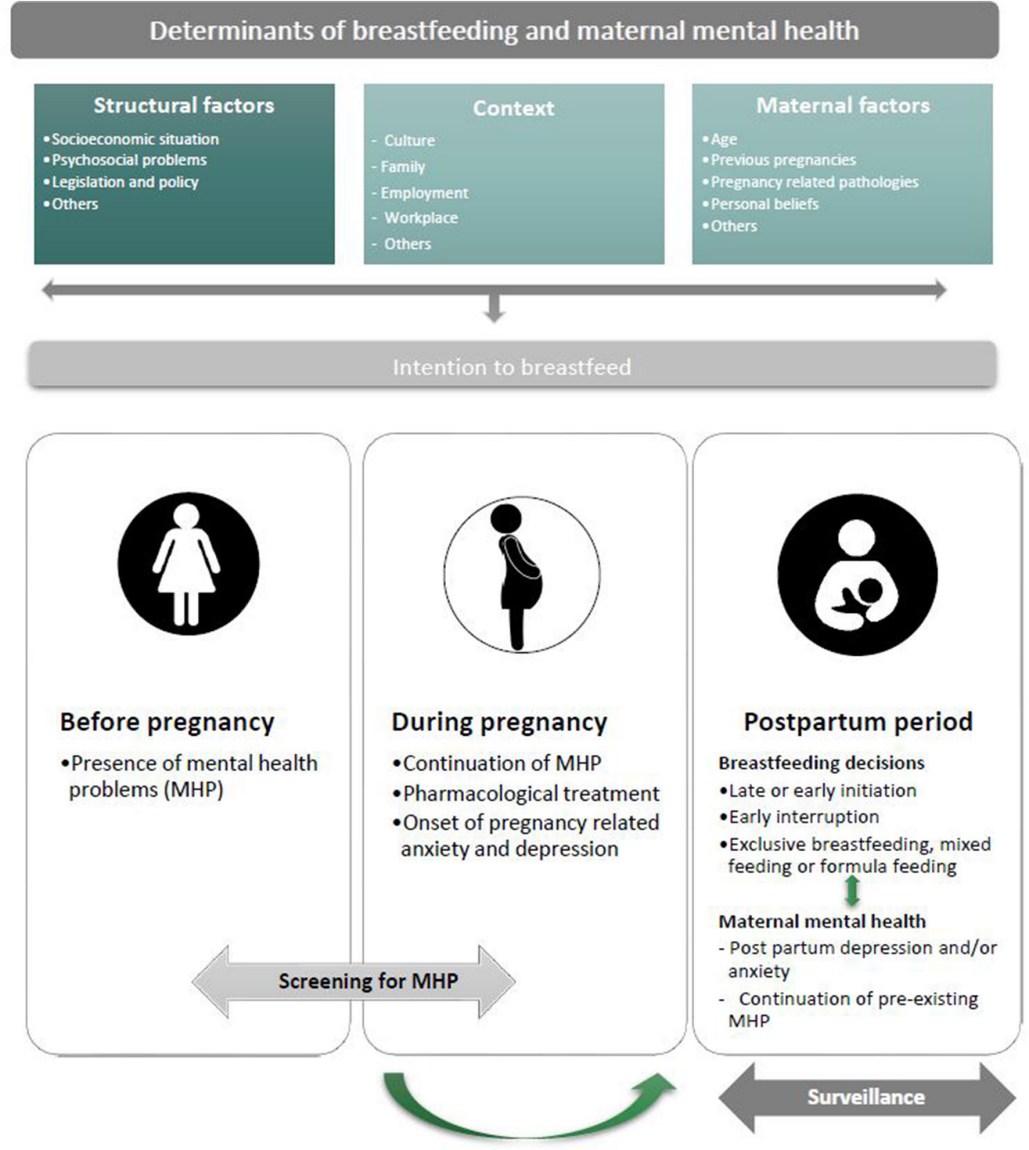
Schematic picture of the complex relationship between breastfeeding and maternal mental health. Working with women in mind means above all considering the main determinants of breastfeeding and maternal mental health: (1) structural factors (i.e., socioeconomic situation, psychological problems legislation and policies, etc.), (2) context (culture, family, employment, workplace, and others), and (3) maternal factors (i.e., age, previous pregnancies, pregnancy related pathologies, personal beliefs, etc.). These domains synergistically influence the maternal intention to breastfeed that, in turn, is set off by the mother's state of mind. Operating with women in mind also means recognizing and isolating the domain(s) most central to overall mental health problems (MHP) and risks, thus, improving successful breastfeeding. Hence, it is fundamental to screen for the presence of early signs and symptoms of maternal depression and/or anxiety before and during pregnancy which puts doctors, midwifes, and mental health experts in a better position to understand the strengths and vulnerabilities of pregnant women who intend or not to breastfeed, assisting them with a mother focused and mother-supportive approach. This, in turn, will allow the timely identification, management, and referral of maternal mental health related complications during the post-partum period, which is intrinsically connected with the decisions to breastfeed. Therefore, the mother's mental health should be among the prime considerations when recommending starting, continuing, or discontinuing breastfeeding. Such a comprehensive approach to breastfeeding will allow women to decide with their mental well-being and consequently, that of their children in mind. Finally, a continuative surveillance of maternal mental health during the post-partum period will consent an early response to the needs of each woman and the development of an individualized approach to how best feed her child and mitigate possible mental health related problems.

A second important factor surrounding the choice to breastfeed is that it is often made in contexts in which the mother does not truly has a choice, such as when medical conditions of mother and neonate do not allow it. Perhaps in these situations the better approach would be to counsel and assist the mother, take away her uncertainties, anxieties, and doubts where possible and prepare her for alternatives conveying the idea that “fed is best” instead of “breast is best” (Figure 1).

## Recommendations

### Screen and Surveil

Routine pregnancy check-ups and postpartum visits (20) represent an ideal time for screening of the presence of early signs and symptoms of maternal depression and/or anxiety and screening tools that include simple questions regarding the intention to breastfeed are most informative and predictive of later problems.

### Identify

Timely identification, management, and referral of mental health related complications in women during the perinatal period is fundamental and by implementing a multidisciplinary approach, which includes perinatal mental health experts, we will be better equipped to understand what mothers want by respecting her and demonstrating a non-judgmental mindset. To do that, we would need to listen more and better.

### Intervene and Prevent

With this information, doctors, midwifes, and mental health experts, will be in a better position to understand the strengths and vulnerabilities of pregnant women who intend or not to breastfeed and intervene accordingly as maternal prenatal anxiety and depression are more readily affected by efficient treatment. Early response to the needs of each woman and the development of an individualized approach to how best feed her child will mitigate possible mental health related problems. In our opinion this is what most women want: a truly comprehensive approach to breastfeeding.

## Author Contributions

VR wrote the first draft. VR and GP performed the literature search. JB was responsible for the concept and the final draft of the paper. All authors contributed important scientific information to the paper, read, and approved the final paper for submission.

## Conflict of Interest

The authors declare that the research was conducted in the absence of any commercial or financial relationships that could be construed as a potential conflict of interest.
